# Substructure-activity relationship studies on antibody recognition for phenylurea compounds using competitive immunoassay and computational chemistry

**DOI:** 10.1038/s41598-018-21394-x

**Published:** 2018-02-15

**Authors:** Fuyuan Zhang, Bing Liu, Guozhen Liu, Yan Zhang, Junping Wang, Shuo Wang

**Affiliations:** 1 Key Laboratory of Food Nutrition and Safety, Ministry of Education of China, Tianjin University of Science and Technology, Tianjin, 300457 China; 2 Beijing Advanced Innovation Center for Food Nutrition and Human Health, Beijing Technology & Business University (BTBU), Beijing, 100048 China; 3 Department of Molecular Sciences, ARC Centre of Excellence in Nanoscale Biophotonics (CNBP), Macquarie University, North Ryde, 2109 Australia

## Abstract

Based on the structural features of fluometuron, an immunizing hapten was synthesized and conjugated to bovine serum albumin as an immunogen to prepare a polyclonal antibody. However, the resultant antibody indicated cross-reactivity with 6 structurally similar phenylurea herbicides, with binding activities (expressed by IC_50_ values) ranging from 1.67 µg/L to 42.71 µg/L. All 6 phenylurea herbicides contain a common moiety and three different substitutes. To understand how these three different chemical groups affect the antibody-phenylurea recognition activity, quantum chemistry, using density function theory (DFT) at the B3LYP/6-311++ G(d,p) level of theory, was employed to optimize all phenylurea structures, followed by determination of the 3D conformations of these molecules, pharmacophore analysis, and molecular electrostatic potential (ESP) analysis. The molecular modeling results confirmed that the geometry configuration, pharmacophore features and electron distribution in the substituents were related to the antibody binding activity. Spearman correlation analysis further elucidated that the geometrical and electrostatic properties on the van der Waals (vdW) surface of the substituents played a critical role in the antibody-phenylurea recognition process.

## Introduction

Phenylurea herbicides constitute an important group of herbicides extensively utilized for herbaceous and perennial weed control in non-crop areas and for the preemergent treatment of fruit crops^1^. Globally, phenylurea herbicides have been detected in surface water^2^, groundwater^3^, soil and sediment^4^ in areas where their extensive use occurred. Phenylurea herbicides are relatively long-lived in the environment, and their introduction to the food chain^5^ via the environment is deemed a serious risk to human health^6^. Therefore, it is evident that environmental risk and food safety problems need to be mitigated.

Currently, immunoassays, such as enzyme-linked immunosorbent assay (ELISA), have been widely used in practice for the fast monitoring of pesticide residues in environmental or food samples^7,8^. However, individual small molecules always belong to one class of compounds, for instance, the phenylurea herbicide family^9^, and most of the resultant antibodies elicited by one specific hapten carrier usually cross-react with other structural analogues^10,11^. This is particularly true if they share an identical or very similar epitope with the hapten used. The mechanism of the cross-reaction between antibodies and different analogues is difficult to elucidate. Although many studies have suggested an antibody recognition mechanism based on X-ray graphs of the antibody with a hapten, crystallization of the title antibody-hapten complex (particularly in polyclonal antibodies) has become a new challenge due to stringent experimental conditions^12–14^.

Since the 1990s, computational chemistry, such as quantum chemistry and molecular mechanics methods, has been used for preliminary guidance on immunoassay development^15–17^. Computational chemistry not only provided a powerful method to explain the cross-reactivity but also became a useful tool to understand the hapten-antibody recognition mechanism in the absence of antibody physicochemical information. In particular, 2D-QSAR or 3D-QSAR methods, such as CoMSIA and CoMFA Contour, have been used in many studies to investigate the binding activities of polyclonal^14,18,19^ and monoclonal^20,21^ antibodies against structurally similar compounds. These methods, however, are only suitable for broad-specific antibodies that can recognize a large number of targets and are not suitable for antibodies that only recognize several compounds^22^.

In this study, a specific hapten was synthesized and conjugated to bovine serum albumin, as the immunogen, to produce a new anti-fluometuron polyclonal antibody. However, when we used the resultant antibody to develop a competitive ELISA, we encountered a general issue, as in most other immunoassays: the specific antibody could also recognize five other analogues with different activities. To explore how this antibody, elicited by one specific immunogen, could cross-react with other targets, the half maximal inhibitory concentration (IC_50_) values, presented as the binding activity of the antibody, were calculated and analyzed. Subsequently, quantum chemistry and molecular mechanics methods were employed to explain how the substituents of the six phenylurea compounds caused the differences in binding activity. Lastly, the molecular surfaces were quantitatively analyzed to correlate the binding activities and the molecular surface descriptors of substituents in the phenylurea compounds.

## Results

### Antibody binding activity analysis

**Figure 1:**
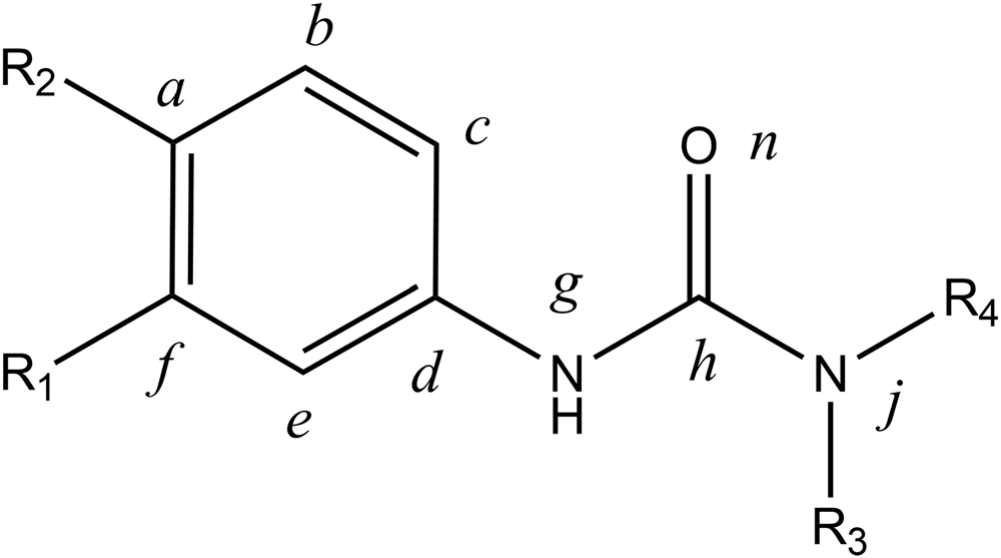
Common structure of the phenylurea molecules in this study. R_1_, R_2_ and R_3_ represent different chemical groups in the different molecules, and R_4_ represents the methyl group. *a-j* represent the number of atoms in the molecule.

According to the two-dimensional structures of the phenylurea molecules (Fig. 1), the 6 title compounds were divided into three groups. The first group consisted of fluometuron and fenuron, which have different substitutes at the R_1_ position. The second group contained neburon and diuron, which have different substitutes at the R_2_ position. The third group included, chlorbromuron and linuron, which have different substitutes at the R_3_ position.

We performed a direct competitive ELISA to evaluate the binding activity of the resultant antibody. IC_50_ and cross-reactivity (CRs) values were calculated to represent the binding activity and specificity of the antibody. These values are shown in Table 1.

**Table 1:** Structures, IC_50_ and CRs of the title phenylurea compounds. The antibody (with a better titer and cross-reactivity, 80000 U and 83.1%, respectively) obtained from number II rabbit was used to establish the ELISA method.

Analytes	R_1_	R_2_	R_3_	IC_50_ (µg/L)	CRs (%)
Fluometuron	-CF_3_	-H	-CH_3_	1.67	100.00
Neburon	-Cl	-Cl	-CH_2_CH_2_CH_2_CH_3_	2.41	69.40
Diuron	-Cl	-Cl	-CH_3_	8.35	19.99
Chlorbromuron	-Cl	-Br	-OCH_3_	9.77	17.10
Fenuron	-H	-H	-CH_3_	11.61	14.38
Linuron	-Cl	-Cl	-OCH_3_	42.71	3.91

The antibody with the best sensitivity to fluometuron (IC_50_ = 1.67 µg/L) was used as the hapten. Although the structure of fenuron and fluometuron are identical except for the substituted group at R_1_, the IC_50_ value of fenuron (11.61 µg/L) is obviously different from that of fluometuron, and the specificity dropped dramatically from 100% (for fluometuron) to 11.61% (for fenuron). This indicates that the lack of -CF_3_ group at the R_1_ position limited the antibody recognition of fenuron. In the second group, neburon and diuron have very different structures from fluometuron, but the antibody has the ability to sensitively recognize them with IC_50_ values of 2.41 µg/L and 8.35 µg/L, respectively. The butyl group at the R_2_ position in neburon is replaced by a methyl group in diuron, and this difference led to a more than 7-fold decrease in the binding activity. In the third group, the replacement of the chlorine atom by a bromine atom at the R_2_ position in chlorbromuron (IC_50_ = 9.77 µg/L) increased the antibody-chlorbromuron binding activity dramatically. In addition, the comparison of the binding activity to linuron (IC_50_ = 42.71 µg/L) and diuron (IC_50_ = 8.35 µg/L) suggested that the different groups at the R_3_ position, where a methoxy group replaced the methyl group, could cause an obvious decline in antibody activity for linuron.

**Figure 2:**
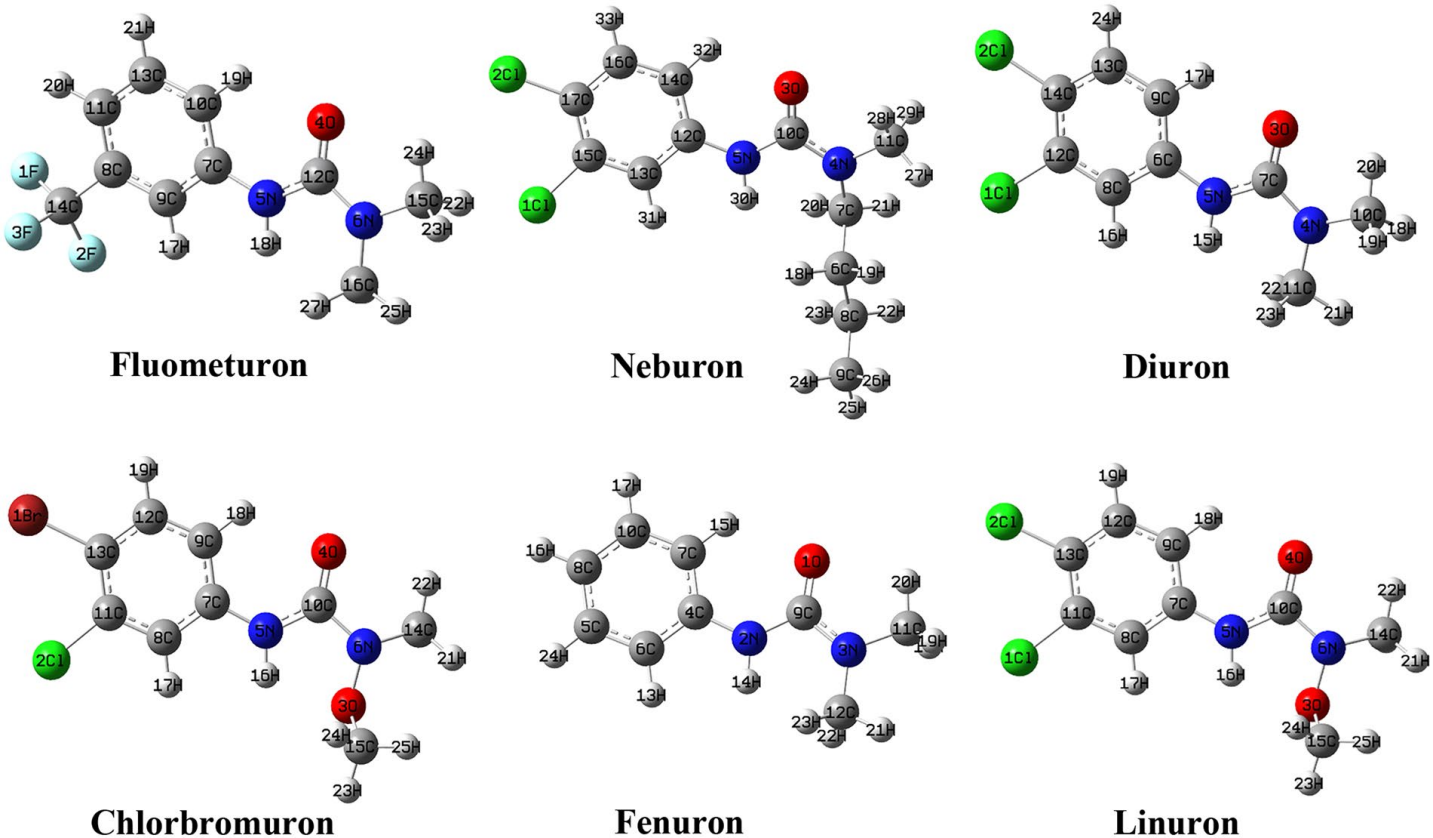
The optimized structures of phenylurea molecules at the DFT/B3LYP/6-311++ G(d,p) level of theory.

The ELISA results demonstrated that the differences in the hapten-antibody binding activity may be caused by the different substitutes in the otherwise structurally similar analytes. However, we cannot explain how different substitutes affect the binding ability just by examining the two-dimensional structural formulas. In the hapten-antibody recognition process, the 3D configuration of the hapten molecule should match that of the active pocket of the antibody, according to the geometric configuration matching mechanism^13,17,23^. Cross-reactions will occur if molecules have the same or similar geometric configuration as the hapten. To understand the recognition mechanism, the 3D configuration of phenylurea molecules were optimized (Fig. 2) and studied by employing quantum chemistry and molecular mechanics methods.

### Conformational analysis and phenylurea molecules alignment

**Figure 3:**
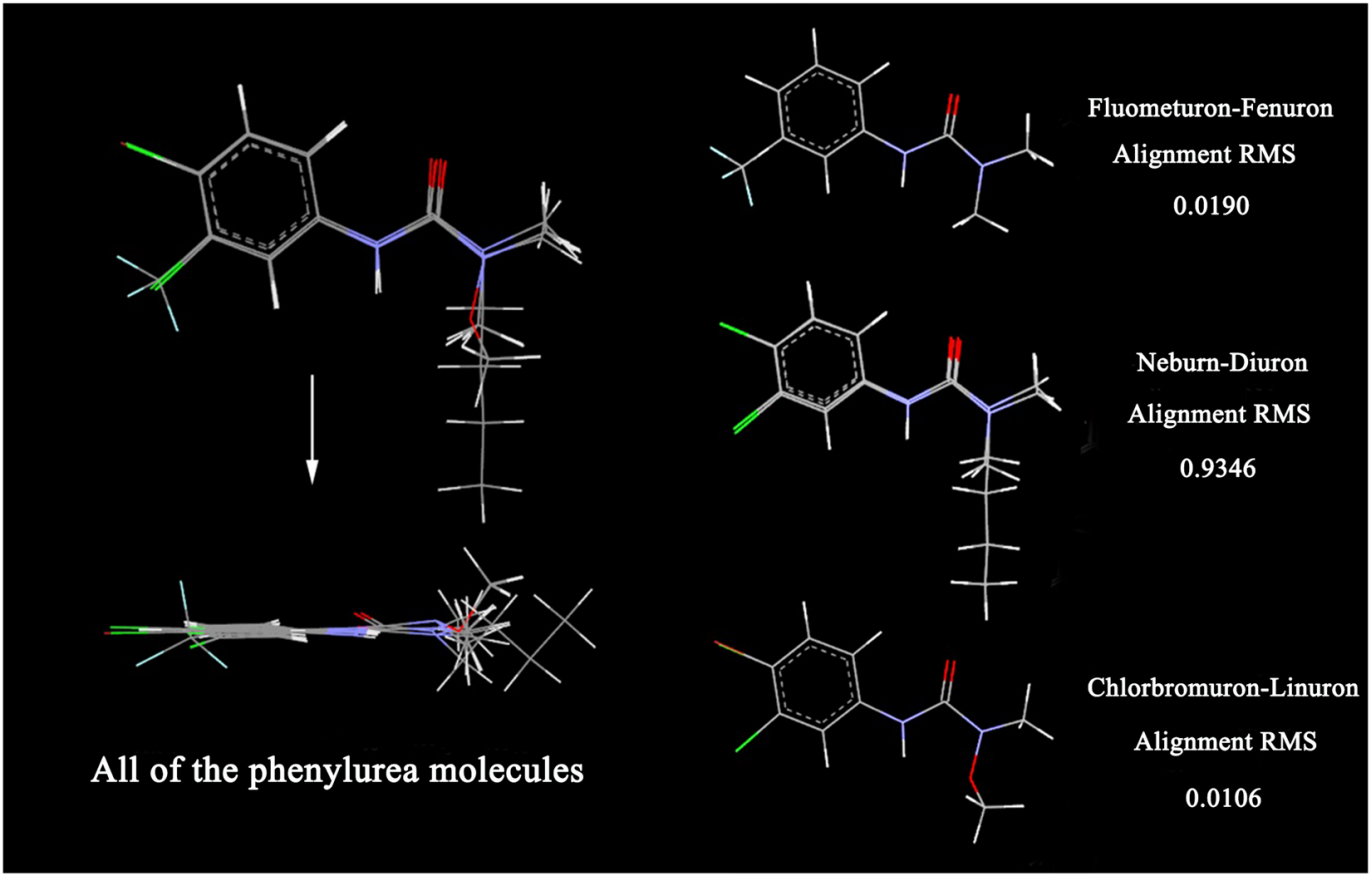
The superposition graph of the phenylurea molecules. Gray sections represent carbon atoms, white represent hydrogen atoms, light blue represent fluorine atoms, blue represent nitrogen atoms, green represent chlorine atoms, red represent oxygen atoms and brown represent bromine atoms.

As seen from the optimized structures in Fig. 2, all molecules have similar bond lengths, bond angles and dihedral angles from atom *a* to atom *j*. Thus, they are well superposed based on their common structures, and the difference in the geometric configuration is mainly located in the different substitutes. Moreover, we can see that molecules in each group have a high degree of alignment based on their common structures (Fig. 3). In the first group, the fluometuron molecule has a very high fitting degree with fenuron based on their common structure (atoms *a-j*, R_2_ and R_3_), showing an alignment RMS value of 0.0190. Even so, the -CF_3_ group at the R_1_ position in fluometuron caused the carbon atoms on the phenyl ring to slightly bend out of the phenyl ring plane. This change contributes to an almost 7-fold gap in the IC_50_ value determined from the geometric configuration. We speculate that the antibody raised by fluometuron contained a -CF_3_ complementary cavity, and this cavity could spatially recognize certain bulky groups, such as -CF_3_.

For neburon, the existence of the bulky butyl group at the R_3_ position also dramatically changed the general conformational structure that was exposed to the antibody, especially the dihedral angle of C_10_-C_5_-C_12_-C_13_ (−164.4°). This value is obviously smaller than those of the other 5 molecules (are all more than 174.0°), and the steric hindrance of the butyl group caused the whole neburon molecule be poorly aligned with the diuron molecule (the alignment RMS value was only 0.9346). Furthermore, neburon shows a better binding ability than diuron. For the third group, the alkoxy group at the R_3_ position in the chlorbromuron and linuron molecules also affects the spatial conformation of the methyl group at the R_4_ position. Chlorbromuron has a high degree of alignment with linuron, and the alignment RMS value is 0.0106. However, the replacement of the chlorine atom by a bromine atom at the R_2_ position resulted in the bond angles of C_1_-C_13_-C_11_ (122.2°) and C_2_-C_11_-C_13_ (121.9°) in chlorbromuron being larger than those in linuron. This scenario contributes to an almost 4-fold activity gap in the binding activity. The geometric configuration analysis shows that certain bulky chemical group at the R_1_ and R_3_ positions play a positive role the antibody-phenylurea binding process. Moreover, the antibody binding sites can also accurately recognize variations of the common structures of these molecules, resulting in differences in binding activity.

### Pharmacophore model analysis

**Figure 4:**
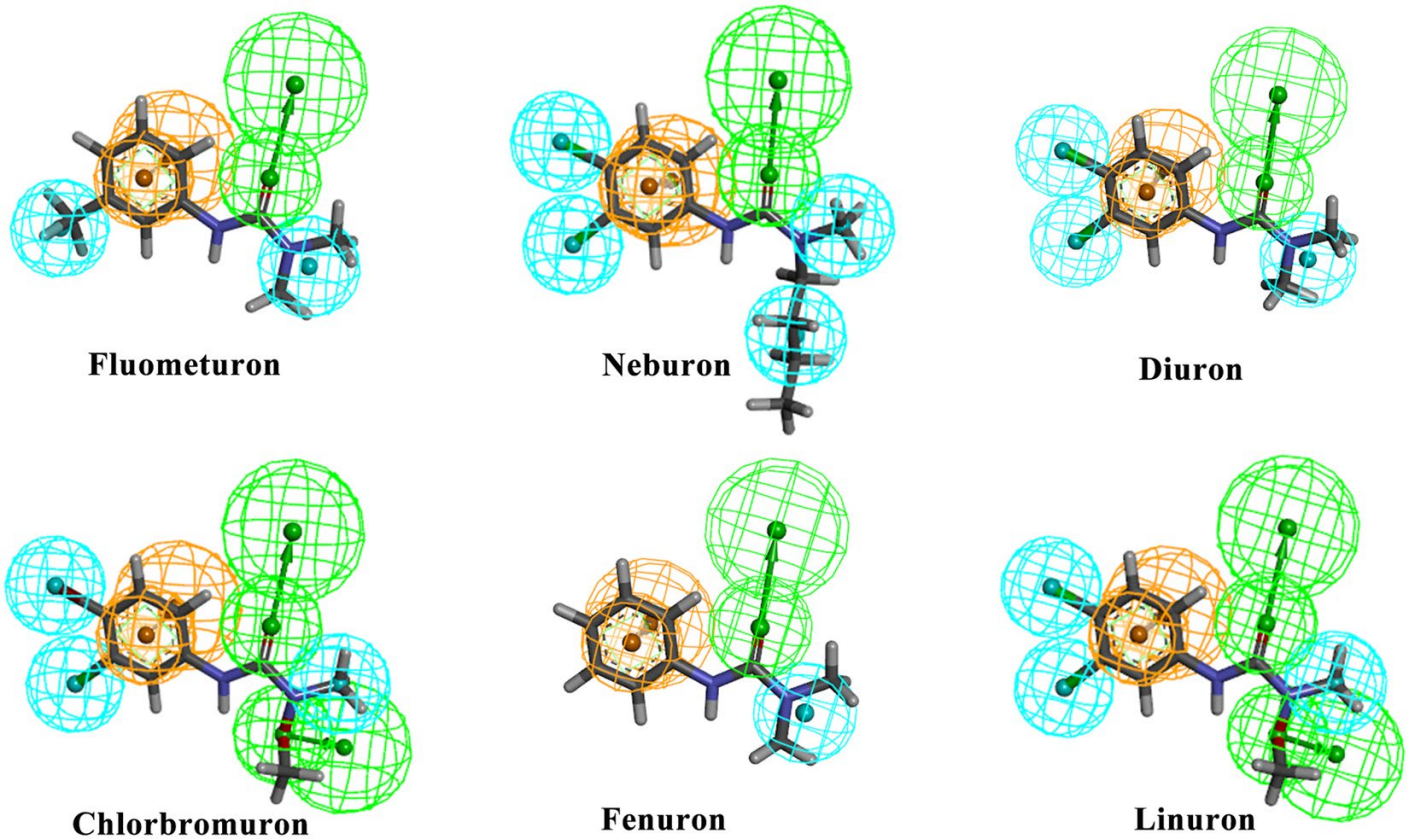
The pharmacophore chemical features mapped onto the phenylurea molecules. The chemical features are color coded, with light blue representing hydrophobic features, orange representing ring aromatic features and green representing hydrogen-bond acceptors.

Fig. 4 shows the pharmacophore models of the phenylurea molecules. The pharmacophore is an ensemble of steric and electronic features of the molecule, such as hydrophobic centroids, hydrogen-bond acceptors or donors and aromatic rings, which are necessary for molecular recognition of a ligand by a biological macromolecule^24,25^. The pharmacophore model of each phenylurea molecule had an aromatic ring feature in the phenyl ring area, a hydrophobic centroid at the R_4_ position and a hydrogen-bond acceptor at the carbonyl group position. The -CF_3_ group at the R_1_ position in fluometuron serves as a hydrophobic centroid. Simultaneously, the chemical features of neburon indicate that it cannot align well with diuron, though their only difference is located at the R_3_ group; the hydrophobicity of the butyl group may influence the steric configuration of the whole neburon molecule, resulting in a higher activity (IC_50_ = 2.41 µg/L) than that of diuron. The chlorbromuron molecule has the same pharmacophore features as linuron but with a bromine atom, and all chlorine atoms serve as hydrophobic centroids. However, we cannot offer a reason for the different activities based simply on pharmacophore features. Except for the R_3_ group, linuron has the same structure as neburon, but the oxygen atom in the R_3_ group in the linuron molecule serves as a hydrogen-bond donor. This led to a decrease in activity, as suggested by the pharmacophore features.

There are some limits in exploring the activity differences according to the pharmacophore analysis. For example, the bromine atom in chlorbromuron and the chlorine atom in linuron serve as hydrophobic centroids, yet we cannot distinguish the differences between them. Furthermore, the hydrogen-bonding and hydrophobic interactions were essentially caused by the electron distribution on the molecular surface^26^. In this way, ESP is also an important and indispensable parameter in the study of the hapten-antibody recognition mechanism.

### Molecular electrostatic potential analysis

The ESP has a unique role in the prediction and analysis of molecular recognition and is often helpful in demonstrating non-covalent molecular interaction properties^27,28^. By employing the ESP surface, we can determine the spatial regions in the molecular structure at which the molecular electrostatic potential is negative or positive. This helps visualize charged regions of a molecule and is qualitatively useful in the analysis of electrostatic interactions between the antibody and the title compound^13,29^.

**Figure 5:**
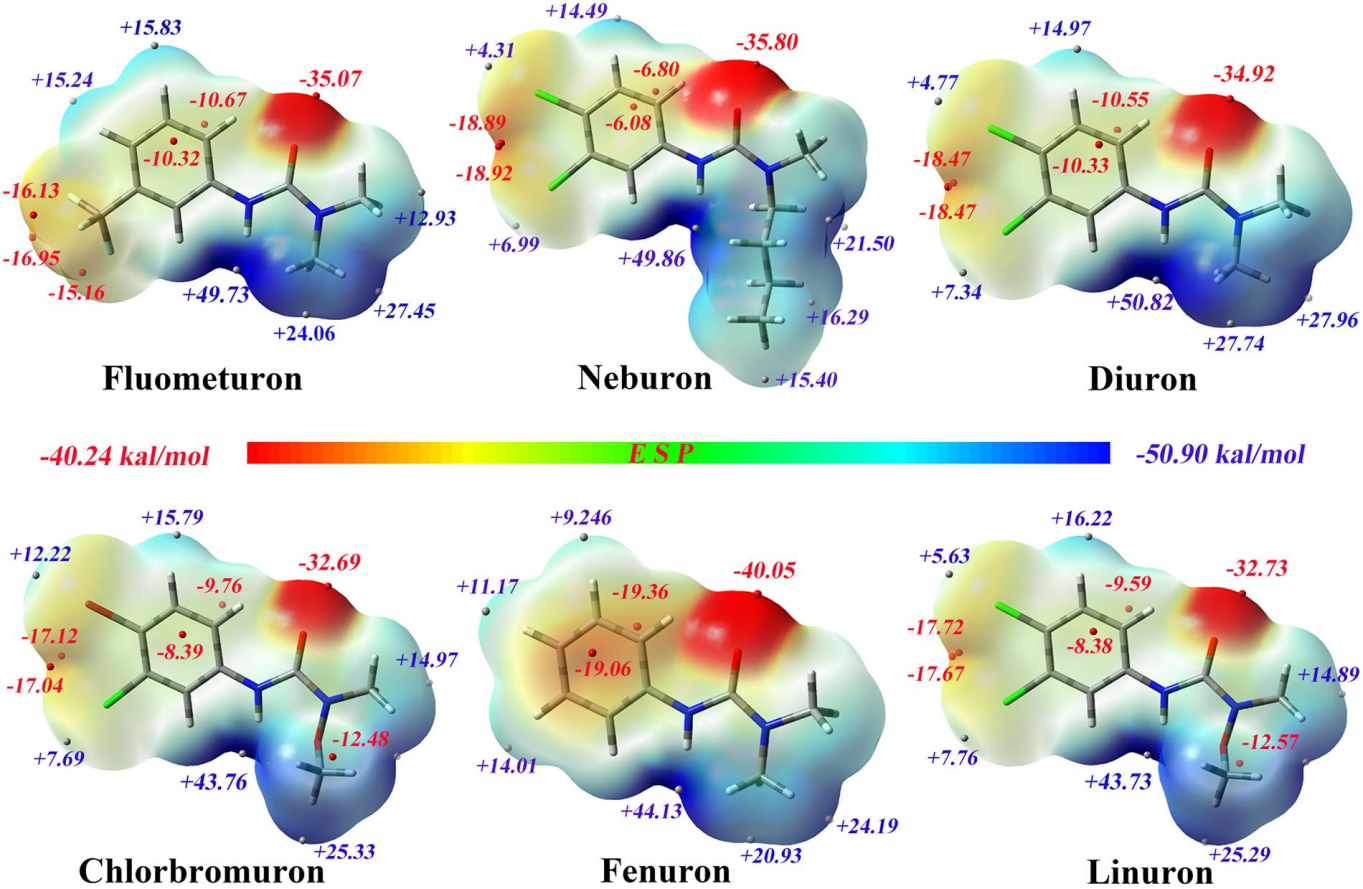
The structures optimized by DFT and the ESP of the phenylurea molecules on the 0.001 a.u. contours of the electronic density of the molecules. The negative ESP regions are indicated in red, and the positive regions in blue. Potential is coded in the following order: red < orange < yellow < green < blue. The red and white spheres represent the minima and maxima of the ESP on the vdW surface.

Three-dimensional plots of the molecular electrostatic potentials of the phenylurea compounds are illustrated in Fig. 5. The ESP surface depicts the phenylurea compound size, configuration, and charge density. The ESP color code ranges from −40.24 to +50.90 kcal/mol. In addition, the minima and maxima of the ESP on the vdW surface were analyzed by Multiwfn and mapped onto the surface of the molecules. All the phenylurea compounds have areas of positive potential at the front of the hydrogen atoms arising from the lower electronegativity of these atoms. This is comparable to carbon or nitrogen atoms, and the most positive potential area (show in dark blue) is located on the surface of the hydrogen atoms associated with nitrogen atoms, where the global maximum point is located. These positive regions can be easily attracted by the negative potential regions of the antibody binding sites. The most negative ESP region (shown in dark red), with a global minimum lower than −32.6 kcal/mol, is located at the front of the carbonyl oxygen atoms. This large negative value is due to the lone pair of electrons on oxygen, which means that this region can be easily attracted by the region of positive potential. The ESP over the phenyl ring carbons is moderately negative, and two minima with similar values are located above and below the ring, reflecting the abundant π electrons of the phenyl ring. As the R_1_ and R_2_ substituents are all electron-withdrawing groups, under the influence of the electron-attracting effect created by groups R_1_ and R_2_, the electron distribution over the phenyl ring also changed.

There are no electron-withdrawing groups associated with the phenyl ring in fenuron, so more electrons are around its phenyl ring than around those of the other five phenylurea molecules. In addition, the minimum values in this area are −19.06 and −19.36 kcal/mol. For fluometuron, the -CF_3_ group, an electron-withdrawing group, attract some electrons around the phenyl ring, resulting in a moderate electron density and three minimum points in front of the R_1_ group, and this -CF_3_ group also led a decrease in the electron density and minimum ESP value around the phenyl ring. We further speculated that the electron-withdrawing group at the R_1_ position could change the electron distribution around the phenyl ring and the R_1_ region, and this was reflected in the higher selectivity for the antibody by electrostatic interaction.

Molecules in the second group have almost identical surface charges, except for in the region of the R_3_ group. As the electron-withdrawing effect is caused by chlorine atoms, there is a high electron density area and two ESP minima between the R_1_ and R_2_ groups. However, the four-carbon chain in neburon changes the electron distribution around the R_3_ position, resulting in a lower electron density and several lower ESP maxima (+15.40 to +21.50 kcal/mol) compared with diuron (electron density of +22.95 and the ESP maxima of +27.74 kcal/mol and +27.96 kcal/mol). This indicates that the alkyl chain could scatter the electron distribution of the R_3_ position and increase the antibody-phenylurea binding activity.

The alkoxy group in linuron reduced the binding activity, possibly because the oxygen atom attracted electrons around it and shared the electrons around the carbonyl group, resulting in a moderate electron density and a minima (−12.57 kcal/mol) around the alkoxy group. Compared with linuron, chlorbromuron also has an alkoxy group at the R_3_ position. However, the chlorine atom at the R_2_ position in linuron has a stronger electron-withdrawing ability than the bromine atom at R_2_ in chlorbromuron. Thus, chlorbromuron has a lower electron density and smaller minima in this area, and the change in the charge distribution may result in the difference in activity.

### Quantitative analysis of the molecular surface

Weak interactions in biochemical systems can be well predicted and explained by analyzing the ESP descriptors on the molecular surface^30,31^. The quantitative analysis of the molecular surfaces in this study is summarized in Table S1. The Spearman correlation analysis between the molecular fragment descriptors and antibody activity (IC_50_ values) is summarized in Table 2. We can see that the parameters – including *S*^+^, *P*, *A*, *Va*^+^ and *B* of the R_1_ group; *B* of the R_2_ group; and *S*^−^, *P*, *I*, *Va*, *Va*^*−*^ and *B* of the R_3_ group – have a positive relationship with the antibody activity, while *S* and *S*^*−*^ of the R_1_ group have a negative relationship with antibody activity. Among these parameters, *B* of the R_1_ group has a key relationship with the antibody activity coefficient, which is significant at the 0.01 level (2-tailed). *S*^+^, *S*^*−*^, *P*, *A* and *Va*^+^ of the R_1_ group also have a crucial relationship with the antibody activity coefficient. All are significant at the 0.05 level. The results showed that the antibody binding activity is linked to geometrical and electrostatic descriptors on the surfaces of the phenylurea molecules, particularly the substituent groups. Specifically, the R_1_ and R_3_ groups played a key role in the antibody-phenylurea binding process.

**Table 2:** Spearman correlation analysis for molecule fragments ESP descriptors and antibody binding ability (expressed by IC_50_ values).

Group	Method	*S*	*S* ^+^	*S* ^*−*^	*P*	*I*	*A*	*Va*	*Va* ^+^	*Va* ^−^	*B*
R_1_	Spearman Correlation	−0.667	0.943**	−0.943**	0.943**	0.314	0.943**	0.314	0.943**	−0.2	0.841*
	Sig. (2-tailed)	0.148	0.005	0.005	0.005	0.544	0.005	0.544	0.005	0.704	0.036
R_2_	Spearman Correlation	0.143	−0.029	0.086	0.314	0.174	−0.029	−0.143	−0.086	0.086	0.600
	Sig. (2-tailed)	0.787	0.9572	0.872	0.544	0.742	0.957	0.787	0.872	0.872	0.208
R_3_	Spearman Correlation	0.257	−0.086	0.671	0.872	0.714	−0.257	0.754	0.257	0.872	0.621
	Sig. (2-tailed)	0.623	0.872	0.215	0.0539	0.111	0.623	0.084	0.623	0.054	0.188

**Correlation is significant at 0.01 level (2-tailed). *Correlation is significant at 0.05 level (2-tailed).

## Discussion

In summary, ELISA analysis confirmed that the obtained antibody could recognize six structurally similar analogs. By analyzing the geometrical conformation and physicochemical properties of the optimized structures, we speculated that the antibody binding pocket could recognize slight differences in the geometric conformation of the molecules. In addition, the negative electrostatic potential of the hydrophobic -CF_3_ group at the R_1_ position and the hydrophobic butyl group at the R_3_ position also contributed to the molecule-antibody binding activity. Meanwhile, the alkoxy group, which served as hydrogen-bond donor, at the R_3_ position decreased the binding activity. Through Spearman correlation analysis between the molecular descriptors and antibody activity, we found that *S*^+^*, S*^*−*^*, P, A, Va*^+^ and *B* of the R_1_ group had key relationships with the antibody activity. The results indicated that the geometrical and electrostatic properties on the vdW surface of the R groups, especially the R_1_ and R_3_ groups, played a key role in the antibody-phenylurea binding activity. This investigation demonstrated that we could explore the antibody recognition mechanism using computational chemistry techniques, despite lacking structural information of the antibody. This study also draws attention to the need to design new haptens to produce specific or broad-spectrum antibodies in the future.

## Methods

### Antibody preparation

In this study, a hapten with a three-carbon spacer arm was synthesized, and then, an immunogen- and enzyme-labeled antigen was obtained by conjugating the hapten to BSA and HRP via the active ester method. The hapten-BSA conjugates were used as the immunogen to immunize two 3-month-old male New Zealand white rabbits (numbers I and II). After the initial immunization and four additional immunizations, the rabbits were sacrificed, and the sera were obtained by centrifugation. Aliquots of the sera were stored at 4 °C in 50% saturated ammonium sulfate. Subsequently, the antibodies were purified by Protein A-Sepharose 4B immunoaffinity chromatography.

### Direct competitive ELISA protocol

The resulting antibodies were evaluated by a conventional direct competitive ELISA method. First, 0.25 µg/well of antibody was coated on a polystyrene 96-well plate using 100 µL/well of coating buffer (0.05 mol/L NaHCO_3_, pH 9.6). After overnight incubation at 4 °C, the excess binding sites were blocked by 200 µL of a blocking solution at 37 °C for 1 h. Then, a mixture of the standard solution and hapten-HRP solution were simultaneously added to each well. After 1 h of competing reaction at 37 °C, substrates A and B were added sequentially for coloration. Finally, the enzymatic reaction was stopped by adding 2 mol/L H_2_SO_4_. The absorbance was determined at a primary wavelength of 450 nm, with a reference wavelength of 650 nm, using a microplate reader. After each step, 250 µL of PBST (10 mM PBS, pH 7.4, 0.05% Tween-20) was used to wash the plate. IC_50_ and cross-reactivity (CR) values were calculated to evaluate the activity of the resulting antibody^13,18,32^.

### Calculation of the antibody binding activity

Inhibition ratios and IC_50_ values were calculated. The inhibition ratio was defined by the following equation:

$$Inhibition\,ratio=\frac{{A}_{Inhibit}-{A}_{Blank}}{{A}_{Control}-{A}_{Blank}}\times 100 \% $$

For this equation, A_Control_ represents the absorbance value of the control group and A_Inhibit_ and A_Blank_ represent the absorbance values of the inhibited group and blank group, respectively. The IC_50_ (µg/L) value was the analyte concentration when the inhibition ratio was 50%.

### Geometry optimization and moleculars alignment

All structures were built in Gaussian 09 ES64L-G09RevE.01 (Gaussian, Wallingford, CT, USA) according to the phenylurea configurations in the Pubchem database. Then, DFT calculations with the B3LYP functional and 6-311++ G(d,p) basis set were performed to optimize the molecules using the Gaussian 09 package^13,33^. There were no imaginary frequencies for all structures, ensuring the local minimum was found. The Molecule Overlay dialog in Discovery Studio 2016 (Accelrys Software, Inc., San Diego, CA, USA) was used to conduct the three-dimensional molecular alignment. The alignment root-mean-square (RMS) value was introduced to measure the degree of molecular superposition in each group.

### Pharmacophore model analysis

The Auto Pharmacophore Generation protocol in Discovery Studio 2016 was employed in this study. This protocol generated ten pharmacophore models for each molecule, and we chose the best one for analysis.

### Molecular electrostatic potential analysis

The Gaussian 09 and Gaussian View 5 packages were employed to conduct the molecular electrostatic potential (ESP) analysis of the title compounds. Minima and maxima of the ESP were generated in the Multiwfn software package and mapped onto the vdW surface.

### Quantitative analysis of the molecular surface

By defining the specific chemical groups (R_1_, R_2_ and R_3_) in the Multiwfn package, descriptors were calculated. Spearman correlation via SPSS software served to detect relationships between molecular descriptors of the molecular fragments of phenylurea and the recognition ability (IC_50_) of a polyclonal antibody.

## Electronic supplementary material

Supplementary information
