# Supplementary material for: Substructure-activity relationship studies on antibody recognition for phenylurea compounds using competitive immunoassay and computational chemistry
Source: Sci Rep. 2018 Feb 15;8:3131. doi: 10.1038/s41598-018-21394-x (PMC5814414; doi:10.1038/s41598-018-21394-x)
Supplement: Supplementary file 1 — Supplementary information [file 41598_2018_21394_MOESM1_ESM.doc]

**Substructure-activity relationship studies on antibody recognition for phenylurea compounds using competitive immunoassay and computational chemistry**

**Fuyuan Zhang^1, 3^, Bing Liu^1^, Guozhen Liu^3^, Yan Zhang^1^, Junping Wang^1^, Shuo Wang^1,2^**

^1^ Key Laboratory of Food Nutrition and Safety, Ministry of Education of China, Tianjin University of

Science and Technology, Tianjin 300457, China.

^2^ Beijing Advanced Innovation Center for Food Nutrition and Human Health, Beijing Technology &

Business University (BTBU), Beijing 100048, China.

^3^ Department of Molecular Sciences, ARC Centre of Excellence in Nanoscale Biophotonics (CNBP),

Macquarie University, North Ryde 2109, Australia.

Correspondence and requests for materials should be addressed to S. Wang. (email:

s.wang@tust.edu.cn).

| **Analytes** | **Fragment** | ***S*** | ***S^+^*** | ***S^-^*** | ***P*** | ***I*** | ***A*** | ***Va*** | ***Va^+^*** | ***Va^-^*** | ***B*** |
| --- | --- | --- | --- | --- | --- | --- | --- | --- | --- | --- | --- |
| **Fluometuron** | R_1_ | 56.00 | 2.00 | 54.00 | 2.87 | 3.74 | -10.85 | 19.50 | 3.49 | 16.01 | 0.15 |
|  | R_2_ | 14.80 | 10.84 | 3.96 | 2.78 | 5.53 | 5.13 | 22.31 | 19.06 | 3.25 | 0.12 |
|  | R_3_ | 37.29 | 37.29 | 0.00 | 0.00 | 3.34 | 22.38 | 19.50 | 21.21 | 0.00 | 0.00 |
| **Neburon** | R_1_ | 32.23 | 3.02 | 29.21 | 3.27 | 4.40 | -8.01 | 23.15 | 3.94 | 19.21 | 0.14 |
|  | R_2_ | 32.47 | 1.67 | 30.80 | 1.42 | 3.77 | -10.76 | 18.02 | 1.55 | 16.47 | 0.079 |
|  | R_3_ | 103.02 | 103.02 | 0.00 | 0.00 | 4.39 | 14.90 | 0.00 | 30.50 | 0.00 | 0.00 |
| **Diuron** | R_1_ | 32.09 | 3.22 | 28.87 | 3.56 | 4.37 | -7.67 | 22.83 | 4.41 | 18.42 | 0.16 |
|  | R_2_ | 32.51 | 1.88 | 30.63 | 1.69 | 3.77 | -10.34 | 17.59 | 1.90 | 15.69 | 0.10 |
|  | R_3_ | 37.25 | 37.25 | 0.00 | 0.00 | 3.42 | 22.95 | 0.00 | 22.16 | 0.00 | 0.00 |
| **Chlorbromuron** | R_1_ | 32.23 | 3.41 | 28.82 | 3.75 | 4.25 | -7.33 | 21.39 | 4.84 | 16.54 | 0.18 |
|  | R_2_ | 36.76 | 5.11 | 31.65 | 6.49 | 4.99 | -7.91 | 26.07 | 12.11 | 13.96 | 0.25 |
|  | R_3_ | 47.92 | 42.50 | 5.42 | 8.96 | 7.23 | 15.42 | 42.29 | 29.41 | 12.88 | 0.21 |
| **Fenuron** | R_1_ | 16.67 | 11.40 | 5.27 | 5.73 | 6.10 | 3.76 | 24.09 | 14.72 | 9.38 | 0.24 |
|  | R_2_ | 16.88 | 9.85 | 7.02 | 5.48 | 6.00 | 0.82 | 23.30 | 8.83 | 14.47 | 0.24 |
|  | R_3_ | 37.32 | 37.27 | 0.00 | 0.09 | 3.55 | 18.79 | 23.28 | 23.19 | 0.09 | 0.00 |
| **Linuron** | R_1_ | 32.17 | 3.46 | 28.71 | 3.85 | 4.26 | -7.24 | 21.77 | 5.00 | 16.77 | 0.18 |
|  | R_2_ | 32.44 | 2.26 | 30.18 | 2.22 | 3.79 | -9.45 | 17.26 | 2.61 | 14.64 | 0.13 |
|  | R_3_ | 47.97 | 42.48 | 5.49 | 9.11 | 7.25 | 15.37 | 42.49 | 29.27 | 13.23 | 0.21 |

**Table S1.** The ESP descriptors determined on the vdW surfaces of the phenylurea molecules.

Weak interaction in biochemical systems can be well predicted and explained by analyzing the ESP descriptors on molecule surfaces. In this study, the quantitative analysis of the molecule surface was summarized in **Table S1**. Of these parameters, the R-group van der Waals surface (*S*) which were considered as the sum of the positive van der Waals surface (*S^+^*) and negative van der Waals surface (*S^-^*) served as geometrical factors. They play an important role in the hapten-antibody recognition process. Besides, the electronic parameters such as the average of ESP over the entire R group surface (*A*) and the internal charge separation (*I*) which is viewed as an indicator of charge separation also has a major impact on the hapten-antibody binding. Moreover, the total R-group ESP variance (*Va*) which can be considered as the sum variance of positive (*Va^+^*) and negative (*Va^-^*) parts was also calculated, and the larger the *Va^+^* and *Va^-^*, the greater the tendency that the molecule interacts with the antibody by positive and negative ESP regions, respectively. Besides, the degree of charge balance (*B*) reflects the variability of ESP, and the closer *B* is to 0.250, the more likely the molecule will interact in a similar way with the antibody molecule through the positive and negative region. The product of *Va* and *B* (*P*) is also a very useful descriptor too; a large value of *P* is indicative of a molecule that has relatively strong tendencies to interact with others of its own kind electrostatically.
